# Protocatechuic Acid Alleviates Dextran-Sulfate-Sodium-Induced Ulcerative Colitis in Mice via the Regulation of Intestinal Flora and Ferroptosis

**DOI:** 10.3390/molecules28093775

**Published:** 2023-04-27

**Authors:** Xuebin Yang, Xin Sun, Feng Zhou, Shuiping Xiao, Lulu Zhong, Shian Hu, Zhe Zhou, Ling Li, Yang Tan

**Affiliations:** 1 Pharmacy of College, Hunan University of Chinese Medicine, Changsha 410208, China; 2 Key Laboratory of Modern Research of TCM, Education Department of Hunan Province, Changsha 410208, China; 3 Liuyang Administration for Market Regulation, Changsha 410399, China

**Keywords:** ulcerative colitis, protocatechuic acid, intestinal flora, ferroptosis, Caco-2 cells

## Abstract

Protocatechuic acid (PCA) is a natural component with multiple biological activities. However, the underlying mechanisms of the effects of PCA on anti-ulcerative colitis (UC) are unclear. A UC mouse model was established by allowing the mice to freely drink a dextran sulfate sodium solution. The mice were administered PCA intragastrically for 7 days. Histological pathology, intestinal flora, and ferroptosis regulators were determined in vivo. Additionally, ferroptotic Caco-2 cells were modeled to investigate the role of PCA in ferroptosis. Our results showed that PCA reduced the levels of the disease activity index, inflammatory factors, and histological damage in UC mice. We also found that the regulation of intestinal flora, especially Bacteroidetes, was one of the potential mechanisms underlying the protective effects of PCA anti-UC. Moreover, PCA downregulated the level of ferroptosis in the colon tissue, as evidenced by a reduced iron overload, decreased glutathione depletion, and a lower level of malondialdehyde production compared with the model group. Similar effects of PCA on ferroptosis were observed in Erastin-treated Caco-2 cells. The results obtained using reactive oxygen species assays and the changes in mitochondrial structure observed via scanning electron microscopy also support these results. Our findings suggested that PCA protected against UC by regulating intestinal flora and ferroptosis.

## 1. Introduction

Ulcerative colitis (UC), a type of inflammatory bowel disease, is a chronic inflammatory disease of the colon with a relatively complex pathogenesis [1]. UC lesions usually involve the colonic mucosa and submucosa and spread throughout the colon [2,3]. In recent years, the incidence of UC has increased worldwide. At present, the clinical treatment of UC is mainly based on improving the intestinal mucosal barrier of patients and reducing the secretion of inflammatory factors [4,5]. However, few specific drugs have satisfactory effects [6].

Maintaining the integrity of the intestinal mucosal barrier is of significance for the prevention and treatment of UC [7]. Previous studies reported that the gut microflora and ferroptosis are associated with the intestinal mucosal barrier in UC [8,9], The intestinal flora acts as a powerful regulator of colonic barrier homeostasis. Typically, the gut microflora affect epithelial repair and mucosal immune responses via metabolic and immune signals during UC pathogenesis. Re-establishing the microecological community is a promising strategy in the search for novel therapies [10].

Dixon proposed the concept of ferroptosis in 2012 [11]. Ferroptosis, which is triggered by iron overaccumulation and lipid peroxidation, is a type of programmed cell death that is different from apoptosis. Mechanistically, ferroptosis is associated with the production of excessive lipid reactive oxygen species (ROS). The biological mechanisms triggering the accumulation of lipid ROS include inhibition of the activity of System Xc-, which is related to the uptake of cystine and depletion of glutathione (GSH), and the inhibition of glutathione peroxidase 4 (GPX4) [12]. A recent report revealed that ferroptosis is related to epithelial cell death in UC [13].

As a common, natural small-molecule phenolic acid compound, protocatechuic acid (3,4-dihydroxybenzoic acid, PCA) often exists in many edible vegetables, fruits, and nuts. The beneficial effects of PCA on human health have been experimentally demonstrated to involve a variety of biochemical mechanisms. The antioxidant, anti-bacterial, and anti-inflammatory effects of PCA have been reported in several studies [14,15,16]. It has been reported that PCA could reduce the oxidative stress caused by dextran sulfate sodium (DSS), reduce the levels of ROS, and improve the activity of antioxidant enzymes, thus preventing oxidative damage to colon tissues [17].

Herein, we induced colitis in mice using DSS, analyzed the effects of PCA in DSS-induced UC, and investigated the underlying mechanisms.

## 2. Results

### 2.1. PCA Ameliorates DSS-Induced Colitis

**Figure 1:**
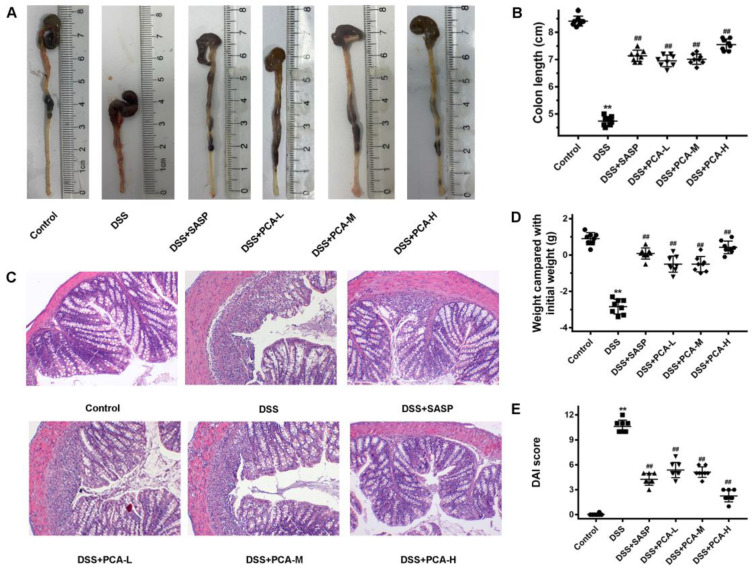
PCA attenuated DSS-induced UC in mice. DSS-induced mice were treated with SASP or PCA. The colon length, (**A**,**B**), changes in body weight, (**D**), and disease activity index (**E**) were determined. The pathological sections the colon tissues stained with hematoxylin and eosin were observed, ×200, (**C**). ** *p* < 0.01, compared with the control group; ## *p* < 0.01, compared with DSS group, *n* = 8.

As shown in Figure 1, at the end of the experiments, the model mice demonstrated significantly decreased body weight and colon length and increased DAI scores when compared with the control group. The administration of PCA or salicylazosulfapyridine (SASP) resulted in an increase in body weight and colon length and decreased disease activity index (DAI) scores when compared with the DSS-treated mice. Furthermore, the results in the DSS+PCA-H group were similar to those observed in the positive group. Histologically, the colonic crypt structure in the control mice was intact, and little infiltration of inflammatory cells was observed. DSS distorted the colonic crypt structure, increased the infiltration of inflammatory cells into the mucosa, caused exfoliation of the goblet cells and epithelial cells, and resulted in a large area of ulcers. As expected, in all treatment groups, the crypt structure of the colon tissues was restored, the infiltration of inflammatory cells was decreased, the goblet cells and epithelial cells were gradually recovered, and the ulcer surface was decreased (Figure 1C).

**Figure 2:**
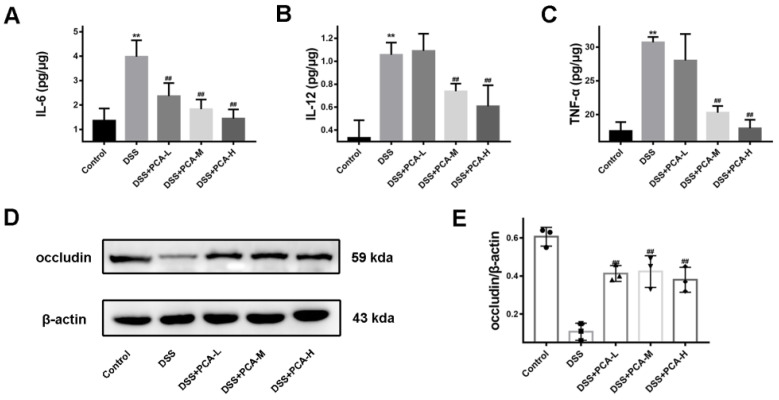
PCA suppressed DSS-induced increase of inflammatory factors and the decrease of occludin protein expression in mice. The concentrations of IL-6 (**A**), IL-12 (**B**), and TNF-α (**C**) in colonic tissue were detected. The expression of occludin protein was determined (**D**,**E**). ** *p* < 0.01, compared with the control group; ## *p* < 0.01, compared with DSS group, *n* = 6 for inflammatory factor detection, and *n* = 3 for occludin protein detection.

In addition, an ELISA analysis of inflammatory factors in colon tissues revealed that treatment with PCA or SASP mitigated the increase in IL-6, IL-12, and TNF-α levels caused by DSS (Figure 2A–C). Consistently, the expression of occludin, a member of the tight junction (TJ) protein family, was significantly downregulated after oral DSS treatment, while treatment with SASP or PCA partially restored occludin expression (Figure 2D,E).

### 2.2. PCA Regulates the Composition of Intestinal Flora 

**Figure 3:**
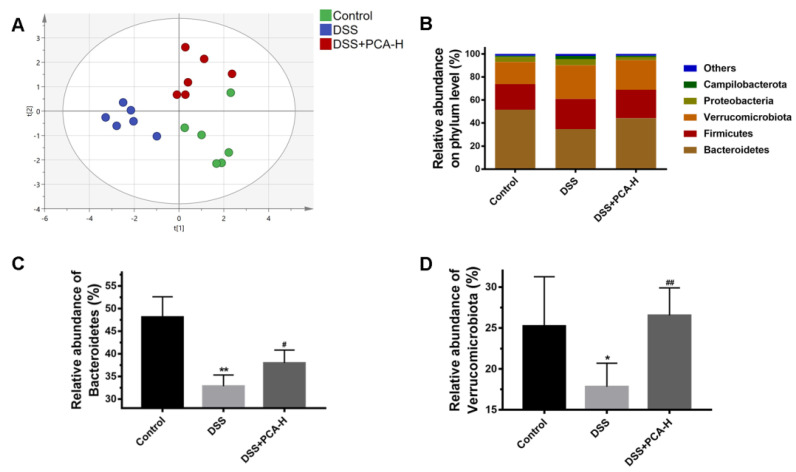
PCA regulates DSS−induced intestinal microbiota disorder in mice. Principal component analysis of gut microbiota at the phylum level (**A**). The cluster of the relative abundance on phylum level (**B**). The relative abundance of Bacteroidetes (**C**). The relative abundance of Verrucomicrobiota (**D**). * *p* < 0.05, ** *p* < 0.01, compared with the control group; # *p* < 0.05, ## *p* < 0.01, compared with DSS group, *n* = 6.

A principal component analysis revealed clustering separated by the relative abundances of gut microbiota at the phylum level among the control group, the model group, and the DSS+PCA-H group (Figure 3A). The values of R2X and R2Y were 31.7% and 17.0%, respectively, indicating that DSS and PCA treatment induced significant changes in the expression levels of the gut microbiota. The relative abundances of gut microbiota at the phylum level are shown in Figure 3B. Firmicutes, Bacteroidetes, and Verrucomicrobiota were the main bacteria in the mice analyzed. Among these bacteria, the relative abundances of Bacteroidetes and Verrucomicrobiota were decreased in the model group compared with the control group, and the treatment of PCA partially restored their relative abundance (Figure 3C,D).

### 2.3. PCA Inhibits DSS-Induced Ferroptosis Injury 

**Figure 4:**
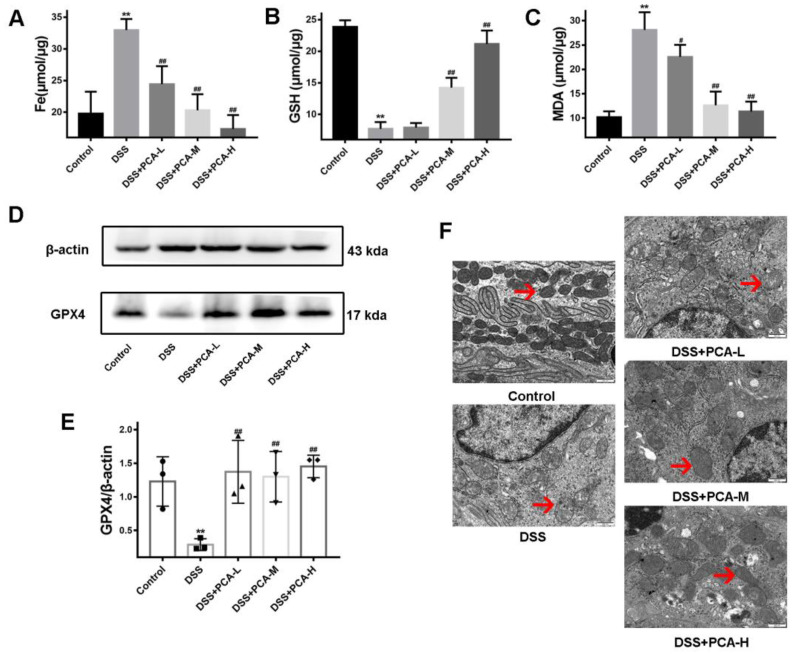
PCA inhibited DSS-induced ferroptosis in mice. The contents of Fe, (**A**), GSH, (**B**), and MDA (**C**) were tested. The expression of GPX4 protein was detected (**D**,**E**). The morphology of mitochondria from colon tissues was observed by transmission electron microscopy (**F**). The red arrows are shown the representative mitochondria in different samples. ** *p* < 0.01, compared with the control group; # *p* < 0.05, ## *p* < 0.01, compared with DSS group, *n* = 6 for Fe, GSH, and MDA detection, and *n* = 3 for occludin protein detection.

As shown in Figure 4, both GSH levels and GPX4 expression were lower, while both MDA and iron levels were higher in the model group than in the control group (Figure 4B). With these results obtained, we confirmed that ferroptosis injury occurred in this DSS-induced UC model. MDA and iron levels were lower, while the expression of GPX4 protein was higher in all three PCA treatment groups compared with those in the model group. Moreover, GSH levels were significantly lower in the DSS+PCA-M and DSS+PCA-H groups compared with the model group. To better observe the inhibitory effect of PCA on ferroptosis, the mitochondrial morphological changes in the colon samples were observed via electron microscopy. Consistent with the trend of the above ferroptosis indicators, the mitochondrial crista in tissues with DSS interference decreased or disappeared and the outer membrane was broken, while PCA protected against the above-described mitochondrial damage, one of the unique morphological characteristics of ferroptosis.

### 2.4. PCA Protects against Ferroptotic Cell Death in Caco-2 Cells

**Figure 5:**
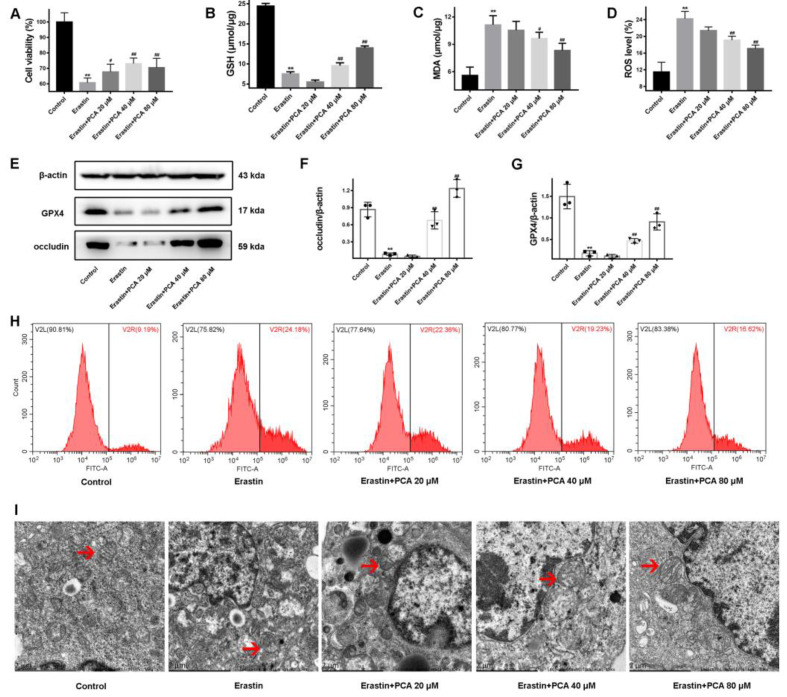
PCA protected against Erastin-induced ferroptotic cell death in cultured Caco cells. Caco-2 cells were administered Erastin to induce ferroptosis in combination with 20 to 80 μM PCA. CCK-8 assay was performed to determine cell viability, (**A**) The contents of -GSH, (**B**) and MDA (**C**) were examined using commercial kits. ROS levels in Caco-2 cells were detected by flow cytometry (**D**,**H**). The expression of GPX4 and occluding protein was detected (**E**–**G**). The morphology of mitochondria from Caco-2 cells was observed via transmission electron microscopy (**I**). The red arrows are shown the representative mitochondria in different samples. ** *p* < 0.01, compared with the control group; # *p* < 0.05, ## *p* < 0.01, compared with DSS group, *n* = 6 for cell viability, GSH, and MDA detection and *n* = 3 for ROS level, occluding, and GPX4 protein detection.

To clarify the role of PCA in regulating ferroptosis, Caco-2 cells were treated with Erastin, a ferroptosis agonist. As expected, ferroptosis was readily induced in the cells by Erastin (Figure 5). Meanwhile, at a concentration between 20 and 80 μM, PCA was added for intervention in this model. A CCK-8 assay revealed that the PCA significantly increased the cell viability of the Caco-2 cells treated with Erastin. Amounts of 40 and 80 μM of PCA increased the expression of occludin protein. Furthermore, PCA treatment not only significantly increased the GSH content and GPX4 protein expression but also decreased MDA and ROS levels, consistent with our results in vivo (Figure 5A–H). As shown in Figure 5I, transmission electron microscopy showed that Erastin induced a decrease in mitochondrial volume and an increase in membrane density in Caco-2 cells. Meanwhile, the mitochondrial crista with Erastin interfering decreased or disappeared, and the outer membrane was broken. As predicted, PCA protected against the mitochondrial damage induced by Erastin, similar to the in vivo result. Altogether, our results suggested that PCA might protect against UC by inhibiting ferroptosis in the colonic epithelium.

## 3. Discussion

UC is a chronic, non-specific inflammatory disease of the colon and rectum. In this DSS-induced experiment animal model of UC, the characteristic features were weight loss, hematochezia, and colon shortening.

PCA is a phenolic acid compound found in various plants. Several studies have already reported the anti-UC activity of PCA [18]; however, few research studies have revealed the its potential mechanisms. In our preliminary animal experiments, we used a broad range of PCA doses to verify the activity of PCA in the treatment of UC. However, when the daily dose of PCA was greater than 30 mg/kg, the mice had more severe hematochezia than the mice in the model group. This made it hard to set PCA at the same concentration (100 mg/kg) as the positive drug (SASP) in a study to better measure its anti-UC effect.

The results of HE staining showed that the samples from the DSS+PCA-L mice had large, ulcerated surfaces, and the ELISA method, used for detecting the concentration of inflammatory factors, IL-12, and TNF-α in colon tissues, showed no significant difference between the DSS+PCA-L group and the model group. Fortunately, PCA showed a good anti-UC effect at doses of 10 and 20 mg/kg with evidence of ethology, pathology, and biochemistry measurements. In the pathological sections of the colonic tissue of mice in the DSS+PCA-H group, few ulcer surfaces and instances of inflammatory cell infiltration were observed. Consistently, mice subjected to 20 mg/kg PCA daily by gavage could better inhibit the production of inflammatory factors in colon tissues compared with the medium dose set in this experiment. In the concentration range set by us, the anti-UC effect of PCA was enhanced with the increase in the dose.

In recent years, the role of the intestinal mucosal barrier in treatment has been studied. The intestinal mucosal barrier is the body’s first barrier for protection against pathogens and other harmful antigens in the intestine. Intestinal epithelial cells (IECs) and TJs form the mechanical barrier, one part of the intestinal mucosal barrier [19].The mechanical barrier can effectively prevent the invasion of intestinal microorganisms, macromolecular toxins, and other antigenic substances [20]. However, during UC, due to the destruction of the mucosal barrier, the permeability of the intestinal mucosa increases. Pathogenic bacteria and endotoxins then migrate to the intestinal lumen, stimulating immune cells in the mucosa lamina propriam finally triggering a strong immune response and aggravating the pathological state of UC [21].

The results of our present study indicate that PCA decreases the histological abnormalities observed in DSS-induced UC. The expression levels of occludin, ZO-1, and claudin-1 are commonly used to evaluate TJ levels in UC. However, previous papers revealed that the expression of occludin is more associated with oxidative stress and ferroptosis [22,23]. In this research, limited by the sample size, only the protein expression of occludin was determined. The disruption of TJs results in damage to the intestinal mucosal barrier, making the gut microbiota more invasive and causing subsequent inflammation. The changes in the gut microbiota during UC could cause the upregulation in the levels of tissue inflammatory factors, such as TNF and IL-6, which also requires further research [24].

Microbiota in the human intestinal tract are estimated to contain as many as 1014 bacterial cells, which is ten times higher than the total human cell population [25]. When UC occurs, due to changes in the intestinal and external environment, the intestinal flora could lose their balance, that is, the abundance of the predominant flora in the intestinal tract is reduced in both type and number, while the abundance of pathogens or opportunistic pathogens increases [26]. Previous studies have shown that probiotics improved the functions of the intestinal mucosal barrier and the immune system, thereby inhibiting the growth of harmful bacteria in the intestine [27].

The role of PCA in regulating gut microbiota was reported previously [28]. In this present research, we observed a significant difference in the phylum classification of the intestinal flora between the model and the DSS+PCA-H groups, indicating that at a dose of 20 mg/kg, PCA at could improve dysbacteriosis and restore the microbiome in the mouse intestine. At the phylum level, the change in the abundance of Bacteroidetes was an important indicator of structural changes of the intestinal flora, which could be used to indicate UC [29]. The present research revealed a significant decrease in the relative abundance of Bacteroidetes in the model group, whereas its relative abundance was increased in the DSS+PCA-H group. In addition, the other microflora affected by PCA, Verrucomicrobiota, has been reported to be associated with the development of obesity and diabetes in a variety of human diseases, although evidence of a direct correlation with UC is still lacking [30]. Thus, we speculate that the effects of PCA in UC were partly related to the regulation of intestinal flora.

Effector molecules or metabolites of some opportunistic pathogens could directly cause the stagnation of IEC proliferation and the disruption of the intestinal mucosal barrier. For example, short-chain fatty acids, especially butyric acid, help maintain the integrity of the epithelial barrier [30]. An imbalance in intestinal flora could decrease the intestinal defense function and increase the levels of pathogenic factors, thus aggravating UC.

Metabolites from the intestinal flora are reported to be involved in the process of ferroptosis [31]. Moreover, the production of short-chain fatty acids is associated with ferroptosis in colon tissue caused by DSS [32]. A recent paper has showed that a secondary metabolite from gut microbiota could alter the level of ferroptosis in intestinal tissues [33]. The exact link between these metabolites and Bacteroidetes is still lacking.

Ferroptosis is a type of iron-dependent programmed cell death which is distinct from apoptosis, necrosis, and autophagy in morphology, biochemistry, and genetics. Ferroptotic death in cells of damaged tissues occurs earlier than apoptosis and other cell death modes [34]. Studies have shown that the initiation of the ferroptosis signal could induce lipid peroxidation in IECs [35]. Meanwhile, it has been reported that in a DSS-induced UC model, intervention with ferrostatin-1, a targeted inhibitor of ferroptosis, could effectively reduce colonic injury and protect the colonic mucosal structure in mice [36].

The two key signals of ferroptosis are the accumulation of iron and lipid peroxidation induced by excessive iron in cells. In cells, iron is usually stored and transported in the form of stable Fe^3+^. The Fe^3+^ entering cells is reduced to Fe^2+^ by reductive metalase. When the cell Fe^2+^ content exceeds a certain limit, ROS are generated by the Fenton reaction. ROS could react with polyunsaturated fatty acids in the cell membrane or organellar membranes by peroxidation, leading to cell death [37].

The core molecular mechanism of ferroptosis is the imbalance between lipid peroxidation damage and antioxidant defense in cells [38]. Two main antioxidant targets involved in ferroptosis are the cystine/glutamate antiporter (System Xc-) and GPX4. Solute carrier family 7 member 11 (SLC7A11) in System Xc- is a transporter protein that takes up extracellular cysteine, which is an important raw material for GSH synthesis. GSH could be oxidized by GPX4 in a way that prevents lipid peroxides from being oxidized, thereby reducing ferroptosis [39]. However, the depletion of GSH could occur as a result of continuous intracellular oxidative stress or impaired cystine transport by System Xc-, leading to the irreversible inactivation of GPX4. The decreased activity or expression of GPX4 could lead to the production of a large number of polyunsaturated fatty acid oxidation products and fatty-acid-free radicals in cells, resulting in ferroptosis [40].

Our study confirmed the induction of ferroptosis in UC due to significant iron overload and oxidative damage in the colons of DSS-induced mice. A previous study has demonstrated the effect of DSS on IEC ferroptosis in vitro [36]. However, other modes of cell death, such as apoptosis, are also induced by DSS [41,42]. Similarily, PCA is also delineated to protect against other forms of cell death, such as apoptosis and pyroptosis [43,44]. To further explain the role of PCA on ferroptosis, Erastin was used as a classical ferroptosis agonist to induce a ferroptosis model in vitro by inhibiting the GPX4 synthesis of GSH and the activity of GPX4. The Caco-2 cell line is often used to measure the effects of drugs on IECs for their epithelial properties [45]. Combined with the changes in mitochondrial structure observed via scanning electron microscopy, we conclude that PCA effectively reduced oxidative damage and prevented Caco-2 cell ferroptotic death induced by Erastin.

According to the existing papers, PCA has two sides for cell protection. High concentrations of PCA have been reported to induce the apoptosis of Caco-2 cells [43]. However, it is also reported that PCA could protect hepatocytes against hydrogen-peroxide-induced apoptosis at concentrations of 25 and 50 μM [46]. Similarily, PCA is also reported to regulate autophagy in different trends [47,48]. In addition to the factor of different PCA concentrations, the use of modeling drugs may also play a role in this.

## 4. Materials and Methods

### 4.1. Reagents

PCA (>98%) was purchased from MACLIN (Shanghai, China). DSS was purchased from Meilunbio (Dalian, China). Occludin (GB111401) antibody was purchased from Servicebio, Inc. (Wuhan, China). GPX4 (AF301385) antibody was purchased from AiFang biological, Inc. (Changsha, China). HRP-conjugated secondary antibody (GAR007-100) was purchased from MultiSciences, Inc. (Wuhan, China). RIPA Lysis Buffer (CW2333) was purchased from CWBIO (Taizhou, China). Tumor necrosis factor-alpha (TNF-α) (70-EK282/3-96) and interleukin-6 (IL-6) (70-EK206/3-96) enzyme-linked immunosorbent assay (ELISA) kits were purchased from MultiSciences Inc. (Hangzhou, China). An interleukin-12 (IL-12) (E-MSEL-M0004) ELISA kit was purchased from Elabscience, Inc. (Wuhan, China). MDA (A003-1) and GSH (A006-2-1) kits were purchased from Nanjing Jiancheng Bioengineering Institute, Inc. (Nanjing, China). An iron assay kit was purchased from Solarbio Life Sciences Institute, Inc. (Beijing, China). An ROS Assay kit (S0033) was purchased from Beyotime Biotechnology Institute, Inc. (Shanghai, China). A BCA detection kit was purchased from NCM Biotechnology, Inc. (NCM Biotech, Suzhou, China), and a CCK-8 kit (C0037) was purchased from Beyotime Biotechnology Institute, Inc. (Shanghai, China).

### 4.2. Animal and Treatment

At 6–8 weeks of age, male C57BL/6 mice were purchased from Hunan Slike Jinda Animal Co., Ltd. (Changsha, China). All mice were reared under specific sterile conditions in the Animal Experiment Center of Hunan University of Traditional Chinese Medicine. All animals received humane care, and the research protocol was approved by the Animal Ethics Committee of the Hunan University of Chinese Medicine (Ethical review NO.: LLBH-202203160756).

After 7 days of adaptive feeding, 48 mice were randomly divided into six groups (*n* = 8 mice per group). Mice in the control group only received pure water every day. Other mice were subjected to the colitis induction protocol through the addition of 3.5% DSS to their drinking water for 7 days. During these 7 days, the mice in the positive control group were subjected to intragastric SASP 100 mg/kg administration every day. Mice in the other three groups (DSS+PCA-L, DSS+PCA-M, and DSS+PCA-H) were treated with 5, 10, and 20 mg/kg PCA every day, respectively. The oral volume of all four treatment groups was 0.2 mL/20 g. Animals in the model and control groups were subjected to intragastric administration of the same volume of saline solution as those in the treatment groups.

### 4.3. Assessment of Ulcerative Colitis Severity

During the experimental period, weight loss, stool consistency, and the degree of colonic bleeding were recorded to calculate the DAI, as previously described by Luo et al. [18]. Additionally, all mice were sacrificed on the 8th day, and the colon specimens were collected for further analysis. Part of the colon tissue was cut into pieces (4 μm) and stained with hematoxylin and eosin (HE) stain.

### 4.4. Measurement of Inflammatory Cytokines in Colonic Tissues

The mice colon samples were homogenized via mechanical homogenization, followed by centrifugation at 1200× *g* for 30 min at 4 °C. The supernatant was collected to analyze the levels of TNF-α, IL-6, and IL-12, using ELISA kits according to the manufacturer’s instructions (Thermo Fisher Scientific, Santa Clara, CA, USA).

### 4.5. Extraction of 16S rDNA and Sequencing

Microbial DNA was extracted from the intestinal digests of six randomly selected mice in the control, model, and PCA-H groups. The total PCR-derived DNA was eluted in the elution buffer. Trimmomatic software (V0.33) was used for quality filtering of the original data. Cutadapt software (V1.9.1) was used for the identification and removal of primer sequences, followed by splicing double-ended reads and removing chimeras via FLASH software (V1.2.11) and UCHIME software (V8.1), respectively. The high-quality sequences were used for subsequent analysis. USEARCH software (V10.0) was used to cluster sequences with 97% similarity. These sequences were assigned to the same operational taxonomic units (OTUs). Combining the species represented by OTUs, the core intestinal microbes in different groups were determined. A principal component analysis was performed to measure the differences among these groups with SIMCA software (V14.1).

### 4.6. Cell Culture and Medicine Induction

The Caco-2 cell line (human colorectal adenocarcinoma cells) was purchased from the Cell Bank of the Chinese Academy of Sciences (Shanghai, China). Caco-2 cells were inoculated into Dulbecco’s modified eagle high-glucose medium containing 20% FBS and 1% double antibody and cultured at 37 °C in 5% CO_2_. Caco-2 cells of passage 5–20 in the logarithmic growth phase were used for the experiments. The cells were treated with 80 μM Erastin for 24 h. At the same time, different concentrations of PCA were added to detect the role of PCA on the level of ferroptosis.

### 4.7. CCK-8 Assay

The cell viability of the Caco-2 cells was determined using a CCK-8 kit. Briefly, 5 × 10^4^ cells were seeded in the 96-well plates, and pharmacological interventions occurred as needed 24 h after cell attachment. Next, the medium was removed, and cells were washed twice with PBS. Then, 100 μL of a new medium containing 10 μL of CCK-8 reagent was added to each well, and after incubation at 37 °C for 1 h, the absorbance of each well was measured at 450 nm with a microplate reader.

### 4.8. Western Blot Analysis

Total proteins were extracted from mouse colon tissues or Caco-2 cells using a RIPA lysis buffer, and the concentrations of proteins were determined via a BCA detection kit. The proteins were separated by SDS-PAGE and transferred to polyvinylidene difluoride membranes. The membranes were blocked with 5% skimmed milk in Tris-buffered saline containing 0.1% Tween-20 (TBST) and incubated with primary antibodies against occludin or GPX4 overnight at 4 °C, followed by incubation with HRP-conjugated secondary antibody for 1 h at room temperature. The immunoreactivity was detected by ECL, and the band intensities were quantified using Image J software.

### 4.9. Measurement of GSH, MDA, and ROS Levels

Protein samples were collected from the mouse colon samples or Caco-2 cells to detect the GSH and MDA contents, using detection kits according to the manufacturer’s instructions. The cells subjected to different interventions were treated with the fluorescent probe DCFH-DA to detect the effects of PCA on the ROS levels in the Caco-2 cells with an ROS Assay Kit by flow cytometry (Beckman, Krefeld, Germany).

### 4.10. Measurement of Iron Contents

Proteins were extracted from colon tissues to determine the iron concentration, using an iron assay kit (Solarbio, Beijing, China) according to the manufacturer’s protocol.

### 4.11. Transmission Electron Microscopy

Colon tissues and Caco-2 cells subjected to different intervention factors were collected, the morphology of the mitochondria was observed via transmission electron microscopy after the following operations. After fixation with a solution containing 2.5% glutaraldehyde for 24 h, the cells were washed in 0.1 M PBS. Then the cells were postfixed with 1% buffered osmium for 2 h, followed by staining with 1% Millipore-filtered uranyl acetate. After dehydration and embedding, the samples were incubated in a 60 °C oven for 24 h.

### 4.12. Statistical Analysis

All data are presented as the mean ± standard deviation (SD). The normal distribution and homogeneity of variance were examined. A one-way analysis of variance was used for data meeting the above conditions; otherwise, the rank sum test was used by IBM SPSS Statistics 21 software. A *p*-value of <0.05 was considered to indicate statistical significance.

## 5. Conclusions

Taken together, we showed that PCA regulated the intestinal flora and inhibited ferroptosis to protect against UC.

## Data Availability

The data generated from the study are clearly presented and discussed in the manuscript.

## Sample Availability

Not available.
